# Mental health assessment during the full-scale invasion within the general Ukrainian population: state, beliefs and behaviors, query to change (cross-sectional study)

**DOI:** 10.1192/j.eurpsy.2024.688

**Published:** 2024-08-27

**Authors:** Y. Korniiko, M. Bajbouj, K. J. Jonas, J. Mueller, S. Kemna

**Affiliations:** ^1^Department of Psychiatry and Affective Neuroscience, Charité University Hospital, Berlin, Germany; ^2^Department of Work and Social Psychology, Maastricht University, Maastricht, Netherlands; ^3^Depratment of Quantitative Methods in Public Health (METIS), EHESP, Paris, France

## Abstract

**Introduction:**

The russian invasion in Ukraine has significantly affected the mental health (MH) of the local population while access to mental health support remains limited due to multiple reasons coming from both the provider and acceptor sides. The war obviously negatively impacts MH but has also paradoxically given an “open window” for shifting current practices both in the healthcare system and within society. Investigation of current people’s attitudes on this matter should be the primary step to address the issue and initiate any change.

**Objectives:**

5 main objectives identified to analyze within the convenience sample were: MH state and self-care behaviors during the full-scale invasion, MH stigma and self-stigma, intention to use professional MH support, beliefs on access to professional MH support, query to change current MH attitudes and practices.

**Methods:**

This research was conducted using primary data collection. The online questionnaire consisted of 5 blocks and was designed based on PHQ-9, DASS-21, PCL-5, Brief-COPE and CAMI. 332 civilians underwent the survey in March-April 2023 and were divided by age, gender, location and situation; inclusion criteria were to be >16 y.o. being affected by war and capable of completing the survey in Ukrainian. Relevant ethical measures were applied. Descriptive and correlational analysis was used to analyze the data.

**Results:**

The majority of respondents rated their mental health as good. Anxiety was the most prevalent emotion, particularly among younger age groups. Different genders and age groups exhibited varying combinations of emotions, such as fatigue, peace, anger, sadness etc. Many participants felt self-reproach for not doing enough; coping strategies varied among age groups. Females were 8.14 times more likely to seek mental health support, and those inside Ukraine were 0.32 times less inclined. 66.2% never seek any MH services, with older men leading; only 8.7% consult specialists during crises, showing gender differences. Distrust in specialist qualifications is one of the barriers on access in people’s beliefs and is more prevalent among older generations. The absence of self-mental health stigma makes individuals 1.91 times more open to accessing support. Location affects openness to change, with Ukraine-based individuals being less open. Lastly, 29.5% consider alternative stress-coping methods, with 40% open to future psychological help.

**Conclusions:**

Our findings show differences in populational attitudes towards MH in Ukraine during the war and therefore the importance of any potential intervention to precisely tailor certain subgroups, beliefs behaviors and needs within them to have a higher chance of being accepted and increase MH support utilization in the population overall.

**Disclosure of Interest:**

None Declared

